# Recent advances in exosome-based immunotherapy applied to cancer

**DOI:** 10.3389/fimmu.2023.1296857

**Published:** 2023-11-07

**Authors:** Jindong Xie, Zihan Zheng, Ling Tuo, Xinpei Deng, Hailin Tang, Cheng Peng, Yutian Zou

**Affiliations:** ^1^ State Key Laboratory of Oncology in South China, Guangdong Provincial Clinical Research Center for Cancer, Sun Yat-sen University Cancer Center, Guangzhou, China; ^2^ State Key Laboratory of Southwestern Chinese Medicine Resources, Chengdu University of Traditional Chinese Medicine, Chengdu, Sichuan, China; ^3^ School of Public Health, Sun Yat-sen University, Guangzhou, China

**Keywords:** exosome, immunotherapy, cancer, immune cells, biomarker

## Abstract

Cancer stands as a prominent contributor to global mortality rates, necessitating immediate attention toward the exploration of its treatment options. Extracellular vesicles have been investigated as a potential cancer therapy in recent years. Among them, exosomes, as cell-derived nanovesicles with functions such as immunogenicity and molecular transfer, offer new possibilities for immunotherapy of cancer. However, multiple studies have shown that exosomes of different cellular origins have different therapeutic effects. The immunomodulatory effects of exosomes include but are not limited to inhibiting or promoting the onset of immune responses, regulating the function of molecular signaling pathways, and serving as carriers of antitumor drugs. Therefore, this mini-review attempts to summarize and evaluate the development of strategies for using exosomes to package exogenous cargos to promote immunotherapy in cancer.

## 1 Introduction

Cancer stands as a prominent contributor to health complications and continues to pose a significant concern within the realm of public health. Treatments for cancer include surgery, radiotherapy, chemotherapy, and molecularly targeted therapies. Combinations of them are commonly used to enhance treatment effectiveness while decreasing side effects (1). However, adverse effects are inevitable, so one hopes to overcome them with new cancer treatment strategies (2). Immunotherapy, which seeks to enhance innate immune responses for the eradication of malignant cells, represents a significant milestone in cancer therapy and has fundamentally transformed the domain of oncology (3). Its main therapeutic approaches include checkpoint inhibitors, over-the-counter cellular therapies, and vaccination strategies (4). Practical applications include immune checkpoint inhibitors (ICIs) such as antibodies targeting programmed cell death 1 (PD-1), programmed cell death ligand 1 (PD-L1), and cytotoxic T-lymphocyte antigen–4 (CTLA-4) (5, 6). Numerous studies demonstrate that exosomes are pivotal in the regulation of tumorigenesis and tumor progression (7, 8).

Exosomes as cell-derived nanovesicles have potential use in cancer immunotherapy due to their immunogenicity and molecular transfer function (9). For example, exosome-based dual delivery biosystems can improve pancreatic ductal adenocarcinoma (PDAC) immunotherapy (10). Another study used ICIs represented by PD-1/PD-L1 immunosuppressants to treat non–small-cell carcinoma (NSCLC) (11). Therefore, this mini-review attempts to summarize the progress made in the application of exosome immunotherapy in cancer.

## 2 The link between exosomes and cancer

Extracellular vesicles are structures that all cells release into their environment, including particles and exosomes (12). Exosomes acquire bioactive molecules, such as nucleotides, proteins, and lipids, from their progenitor cells. It is known that exosomes are derived from endocytosis, which is a pathway that can be used by different types of cells (13). According to their origin, exosomes have different functions (14). It has been found that DC-derived exosomes (DEX), which possess major histocompatibility complex (MHC) I, MHC II, CD86, and HSP70-90 chaperones, thereby facilitating the initiation of CD4+ and CD8+ T-cell activation (15, 16). In contrast, exosomal PD-L1 secreted by tumor cells can achieve immune escape by binding PD-1 on the surface of immune cells (17).

The tumor microenvironment (TME) consists of tumor mesenchyme, and alterations in the signaling network occurring in the TME might influence tumor progression (18). The link between exosomes and TME is precisely reflected in the fact that signaling pathways affected by exosomes can influence TME (19). Thus, TME is one pathway by which exosomes can alter tumorigenesis and progression. Efforts have been undertaken to develop exosomes that specifically target the TME and exert regulatory effects on cancer progression, owing to the considerable potential of exosomes in influencing the TME. For example, Wang et al. revealed that tumor cell–derived exosomes (TDEs) from glioblastoma could fuse to the surface of T cells, increase the concentration of adenosine around T cells, activate adenosine receptor 2A, and inhibit aerobic glycolysis of T cells, thus inhibiting clonal proliferation of T cells and promoting the TME land formation by decreasing energy production (20). Another study found that exosomes derived from prostate cancer can increase the expression of C-X-C motif chemokine receptor 4 (CXCR4) in myeloid-derived suppressor cells (MDSCs) by activating the toll like receptor 2/nuclear factor kappa-B (TLR2/NF-kB) pathway, thereby promoting the migration of MDSCs to the TIME and enhancing the formation of TME (21). Likewise, Liu et al. have demonstrated that lung TDEs can facilitate the formation of TME by enhancing M2 polarization, leading to cancer progression (22).

Moreover, exosomes can influence drug resistance in tumors. For example, exosomes can exert an influential impact on the chemotherapeutic response of cancer cells (23). Comparable effects are likewise observed in the fields of radiotherapy and immunotherapy. Notably, exosomes can help cancer cells achieve immune evasion. It has been demonstrated that in breast cancer, the ability of exosomes to transmit SNHG16 may mediate overexpression of CD73 on Treg cells and lead to immunosuppression (24). Another study shows that PD-1 pathway prevents T cells from proliferating and causes T-cell exhaustion, whereas exosomes can carry PD-L1 components that suppress immunity (25). In addition, exosomes derived from M2 macrophages carrying miR-155-5p induce immune escape by impairing zc3h12b-mediated stabilization of IL-6 and promote colon cancer development (26). Numerous studies have demonstrated a significant association between exosomes and the modulation of the immune system within TME. Therefore, exosome-based immunotherapy may be a breakthrough point in cancer treatment. In conclusion, exosomes have been shown to be a key factor influencing tumor progression and treatment.

## 3 Immunosuppression of exosomes and corresponding therapy

Species capable of suppressing the body’s immune response are present in exosomes, mainly TDEs (12), also including some exosomes derived from non-tumor cells. Exosomes of non-tumor origin included Treg-derived, cancer-associated fibroblast–derived, and myeloid-derived exosomes. This section describes the immunosuppressive function of TDEs and therapeutic approaches. It is widely recognized that, under typical circumstances, antigen-presenting cells effectively display tumor-associated antigens to T cells, thereby stimulating their cancer-killing capacity and consequently exerting a favorable impact on the immune system. Natural killer (NK) cells can also induce cytotoxicity through their recognition of specific molecules present on the surface of tumor cells. However, in response to the immune system, tumor cells have evolved a variety of mechanisms to evade it, such as decreasing immunogenicity and regulating antigen presentation (27). TDEs have been shown to have immunosuppressive effects, promoting tumor growth (28).

TDEs can suppress the immune function of lymphocytes. The modulation of lymphocyte activation is influenced by a sequence of signaling molecules. Negative immune checkpoints, such as PD-1, CTLA-4, and T-cell immunoglobulin-3 expressed on immune cells, have the potential to elicit cellular exhaustion, which might inevitably cause the decline of immune function, weaken the killing effect on tumor cells, and ultimately facilitate immune evasion (29). These checkpoints are actually located on the surface of the tumor cells and are present on the surface of the exosomes secreted by the cells as well (30). Therefore, TDEs have the potential to convey multiple immunosuppressive signals, thereby impeding T-cell proliferation and exerting an influence on tumor immunity. One prominent instance is exosomal PD-L1, which exhibits functional equivalence to cellular PD-L1 and has the ability to engage with PD-1 on CD8 T cells, thereby suppressing their anti-tumor reaction (31). Exosomal PD-L1 achieves immunosuppressive effects by enhancing PD-1/PD-L1–induced inactivation of T cells. In addition, exosomes can inhibit the immune function of other lymphocytes, such as T helper cells (32), B cells (33), dendritic cells (DCs) (34–36), and NK cells (37).

Given that exosomes can carry multiple immunosuppressive signals, it has been shown that TDEs can be used as cancer biomarkers. Exosomes exhibit extensive distribution within the somatic circulation and possess the potential to function as diagnostic markers in the context of “liquid biopsy” for diverse malignancies (38). Because the elevated concentrations of exosomes were observed in the circulation of patients with various malignancies, it is confirmed that tumor progression can be understood by monitoring the concentration of exosomes. In addition, exosomes possess a substantial quantity of distinct nucleic acids, thereby enhancing their efficacy as biomarkers. For instance, previous study has demonstrated a significant decrease in the mRNA expression levels of four specific genes (interleukin-8 (IL-8), transforming growth factor-β (TGF-β), tissue inhibitor of mental protease 1 (TIMP-1), and zeta chain of T cell receptor associated protein kinase 70 (ZAP-70)), within exosomes derived from the bodily fluids of glioma patients undergoing therapeutic interventions (39). Hence, the efficacy of monitoring nucleic acids in exosomes for assessing tumor status and treatment outcomes is evident. In addition, because exosomes secreted by different tumor cells have different compositions, diagnostic differentiation of tumors can be performed by identifying specific exosomes (40). Moreover, one of the advantages of exosomes as biomarkers is that, even if the number of exosomes is too small to be detected, the molecules can be amplified by polymerase chain reaction technology for the purpose of detection. Certain tumor-specific proteins in exosomes are also strongly associated with tumor grading, staging, treatment success, and survival of patients (41). Exosomal PD-L1 serves as the archetypal illustration, and research has substantiated a positive correlation between the presence and magnitude of PD-L1 expression on exosomes derived from tumors in the peripheral blood of patients and the stage of the disease (31). Another study has shown that exosomal PD-L1 levels can be used as a predictor of the effectiveness of immunotherapy in NSCLC when it comes to conveying diagnostic and prognostic information (42). Exosomes can be used as biomarkers through a number of different components, thus indicating cancer progression as well as the outcome of treatment.

However, with regard to counteracting the effects of exosomal suppression of immunity, we still need to find alternative approaches. For instance, exosomal PD-L1 exhibits resistance toward anti-PD-1 therapy and persists in its ability to suppress T-cell immune response, both through direct and indirect means, even in the presence of ongoing antibody treatment (43). Thus, for exosomal PD-L1, exosome clearance may be a feasible and necessary concomitant therapy. It has also been demonstrated in the literature that exosome biogenesis can be inhibited by inhibiting exosome release. Among them, GW4869 is widely used and can be used to treat breast cancer cells, which leads to a reduction in exosome secretion (44), which, in turn, attenuates its immunosuppressive effects and delays tumor progression.

## 4 Exosome for activation of immunity and vectors

DCs are key antigen-presenting cells in the human body and have played an important role as important targets in previous tumor immunotherapy, with the main mechanism being the mediation of tumor immunity through the activation of CD8+ and CD4+ T cells (15). In addition, DCs can secrete exosomes that induce anticancer responses, making them an ideal antigen for DC vaccines (45). DEX containing MHC I, MHC II, CD86, and HSP70-90 chaperones are capable of triggering the activation of CD4+ and CD8+ T-cell activation (16, 46), thereby inducing more effective antitumor immunity and addressing the poor immunogenicity of tumor antigens. Other studies have confirmed that DEX activates CD8+ and CD4+ T cells and induces anti-tumor immune responses via *in vitro* CD80-mediated exosomes and *in vivo* endogenous IL-2 (47, 48). According to previous research, DCs have been employed in tumor immunotherapy in diverse preclinical and clinical investigations. Notably, a study has shown that CD47-mediated exosomes exhibit resistance to phagocytosis by monocytes and macrophages, thereby prolonging the exosomes’ lifespan in the bloodstream. Consequently, the administration of exosomes effectively inhibited cancer growth in various mouse models of pancreatic cancer and significantly improved overall survival rates (49).

The utilization of DC vaccines in tumor immunotherapy exhibits significant promise and has undergone rigorous evaluation through multiple clinical trials. According to research findings demonstrating robust T-cell responses to autologous tumor antigens induced by allogeneic immunoglobulin G (IgG)-loaded DCs, it has been established that DC vaccines can elicit T-cell immunity in patients with melanoma. The main pathway is that it may promote the expansion of a highly diverse neoantigen the T-cell receptor repertoire, thereby indicating promising prospects for their future clinical utilization (50, 51). In addition, the presence of enhanced multifunctional T cells was detected in glioblastoma patients vaccinated with CMV pp65 mRNA-loaded DCs, treated with CMV pp65 RNA-pulsed DCs, suggesting that adjuvant DC vaccination enhances transferred T-cell immune responses *in vivo* (52). In anti-HER2 clinical trials, DC vaccination induced tumor-specific T-cell responses in patients with human epidermal growth factor receptor-2 (HER2)-positive breast cancer. The team found that immune and clinical responses were independent of the route of vaccination, to be refined in subsequent studies (53). The limited efficacy of classical DC vaccines may be mainly due to the presence of multiple immunosuppressive factors in TME. Novel DC vaccine strategies have been actively investigated with the aim of consistently increasing the magnitude of DC cross-presentation and effector T cell–derived antitumor immunity. Biomaterial-based and ICD-induced DC vaccines were found to have unique advantages in recruiting and activating endogenous DCs (54). These studies suggest that novel DC vaccines will be beneficial for DC vaccines to provide a major breakthrough in the field of cancer immunotherapy. In conclusion, DC vaccines will be a new direction for tumor immunotherapy in the future, but the relevant studies and trials need to be further improved.

## 5 Exosomes for targeted therapies and drug resistance in cancer treatment

Exosomes are ubiquitously present in diverse bodily fluids, such as blood and urine, and possess the potential to furnish valuable insights into the cellular or tissue origins of their cargo, thereby aiding in disease diagnosis. Moreover, they exhibit the ability to be internalized by specific cell populations, thereby facilitating the transfer of their contents and enabling intercellular communication (55, 56). Thus, exosomes can be used to load drugs, and their lipid composition and their stable membrane structure ensure carrier stability. Studies have demonstrated the use of exosomal miRNAs as molecular diagnostic markers for tumors, and the use of nanoparticle platforms for transporting miRNAs can be used for targeted therapies for diseases and tumors (57). A study showed that adipose-derived mesenchymal stem cells (MSCs) have full ability to transfer miR-122 via exosomes, thereby sensitizing hepatocellular carcinoma cells to chemotherapeutic agents (58). U251-derived exosomes were potently and specifically incorporated into cancer cells and were taken up by their parental cells independently of surface protein ligands on exosomes, indicating that targeting U251-derived exosomes on cancer cells might help in the development of cancer-targeted drug delivery systems (59). Utilizing exosomes as carriers is still in its infancy, but its potential could make it an attractive and potentially effective tool for drug delivery in future cancer therapy (60).

**Figure 1:**
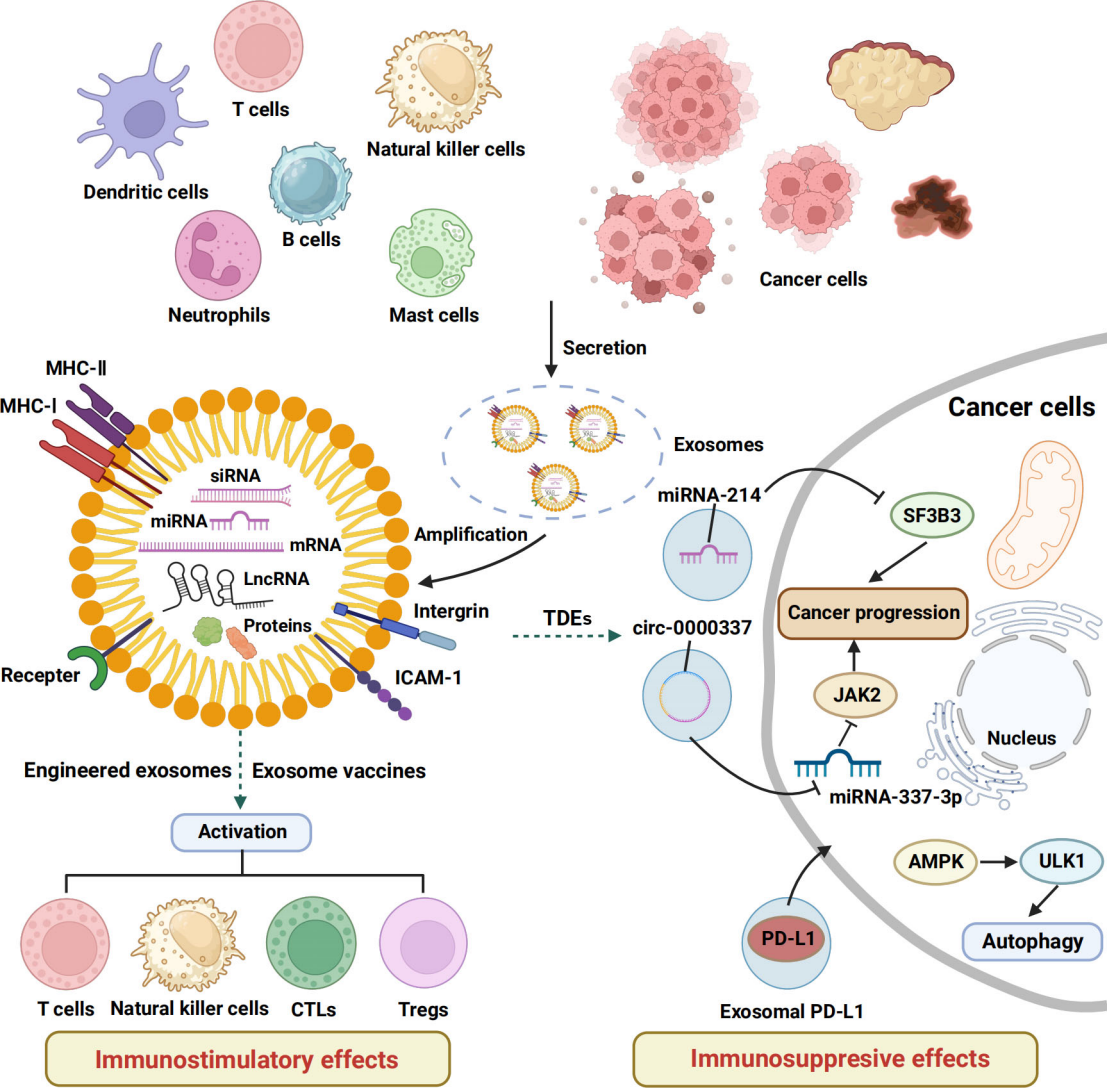
The immunostimulatory and inhibitory effects of exosomes. This schematic shows the basic structure of exosomes and the mechanisms and functions of exosomes released by a variety of cells in regulating the immune response of tumor-bearing hosts. MHC, major histocompatibility complex; ICAM, intercellular cell adhesion molecule; CTLs, cytotoxic T lymphocytes; Tregs, regulatory cells, TDEs, tumor cell–derived exosomes .

MSCs possess the remarkable ability to undergo self-renewal and differentiate into various lineages, thus exhibiting pluripotency (61). Recent studies have shown a significant relationship between MSC-derived exosomes and cancer resistance to chemotherapeutic agents, targeted therapeutic agents, radiotherapy, and immunotherapy (62). Therapeutic resistance is a serious challenge to the cure of patients with cancer, manifested by genetic or phenotypic alterations as well as the emergence of resistance after initial successful treatment (63). In the field of PDAC, a study has substantiated the viability of employing an exosome-based dual delivery biosystem for the purpose of immunotherapy. The utilization of exosomes derived from bone marrow MSCs can effectively enhance tumor targeting, consequently augmenting drug accumulation within the tumor vicinity (10). Exosomes originating from chemotherapy-resistant colorectal cancer cells can transfer ciRS-122 across cellular boundaries, thereby facilitating glycolytic activity and subsequently diminishing the drug sensitivity of chemotherapy-responsive cells (64). MSC exosomes significantly induce resistance to 5-fluorouracil in gastric cancer cells (65). Breast cancer cell–induced MSC-derived exosomes promote breast cancer dormancy, enhance sensitivity to carboplatin, and are associated with carboplatin resistance. This provides a novel, non-toxic therapeutic strategy to target dormant breast cancer cells through systemic administration of mesenchymal stem cells (66). Alterations in bone marrow MSC–derived exosome content are significantly associated with tyrosine kinase inhibitor resistance in acute myelocytic leukemia (67). MSC-derived exosomes can impair protective anti-tumor immunity through up-regulation of PD-L1 in myeloid cells and down-regulation of PD-1 in T cells in breast cancer (68). MSC exosomes overexpressing miR-34c can be used to inhibit the development of tumors and promote the sensitivity of nasopharyngeal carcinoma to irradiation, thereby improving the therapeutic effect of radiation (69). These observed intercellular signaling implies that exosomes hold promise as a prospective therapeutic target for addressing drug resistance in cancer therapy (**Figure 1**).

## 6 Future recommendation and conclusion

Increasing evidence suggests that exosomes have great potential in diagnosis, prognosis, and assessment of tumor efficacy. On the basis of the close connection between exosomes and tumor immunity, exosome-based immunotherapy will be a new direction for cancer immunotherapy in the future. In addition to this, exosomes can promote chemoresistance within the TME and can also be used as endogenous carriers and biomarkers to transmit biological information between cells.

However, to date, most exosome studies have been conducted in cellular experiments, and larger, multicenter, and long-term studies are urgently needed for exosome-based cancer immunotherapy. Tumor cells can evade host immune surveillance by fostering a highly immunosuppressive microenvironment (70), and DCs have been used with limited success as cancer vaccines (71, 72). The crucial factor contributing to the efficacy of DC immunotherapy lies in its capacity to activate T cells, thereby determining the potential of a DC vaccine to induce a robust and enduring anti-tumor immune reaction. It has now been demonstrated that the removal of sialic acid can greatly enhance the DC anti-tumor immune response. Hence, sialidase treatment based on this study sialidase treatment can be used as a powerful tool to increase MHC I expression and enhance antigen presentation of MHC I to improve the effectiveness of therapeutic cancer DC vaccines (73).

In the clinical setting, there are also challenges with regard to the uncertain biosafety of exosomes, and the development of novel and safe techniques for extracting and isolating exosomes is key to ensuring the safety of exosome therapy (74). How to improve the stability of exosomes *in vivo* is also a major challenge, and the optimal dosage, drug distribution, therapeutic routines, and biosafety of exosomes in cancer therapy need to be better evaluated, and long-term monitoring platforms are needed for clinical use. The development of more efficient and cost-effective methods for exosome extraction and isolation is also important for the clinical application of exosome-based cancer immunotherapy. Despite the difficulties and challenges, exosome-based cancer immunotherapy remains promising.

## Author contributions

JX: Data curation, Writing – original draft. ZZ: Data curation, Writing – original draft. LT: Data curation, Writing – original draft. XD: Validation, Writing – review & editing. HT: Validation, Writing – review & editing. CP: Conceptualization, Funding acquisition, Supervision, Writing – review & editing. YZ: Conceptualization, Supervision, Writing – review & editing.
